# Evaluation and characterization of anti-RalA autoantibody as a potential serum biomarker in human prostate cancer

**DOI:** 10.18632/oncotarget.9869

**Published:** 2016-06-07

**Authors:** Jitian Li, Liping Dai, Ningjing Lei, Mengtao Xing, Pei Li, Chenglin Luo, Carlos A. Casiano, Jian-Ying Zhang

**Affiliations:** ^1^ Department of Biological Sciences, The University of Texas at El Paso, El Paso, TX 79968, USA; ^2^ Henan Key Laboratory of Tumor Epidemiology and Henan Academy of Medical and Pharmaceutical Sciences, Zhengzhou University, Zhengzhou, Henan 450052, China; ^3^ Center for Health Disparities and Molecular Medicine, Department of Basic Sciences, Loma Linda University School of Medicine, Loma Linda, CA 92354, USA

**Keywords:** autoantibody, tumor-associated antigens (TAAs), cancer immunodiagnosis, prostate cancer

## Abstract

Autoantibodies against intracellular tumor-associated antigens (TAAs) are commonly found in human cancers. In this study, we characterized the serum autoantibody response to the RalA, Ras-like GTPase, in patients with prostate cancer (PCa). The autoantibodies were detected by immunofluorescence assay in PCa cell lines, ELISA, and immunoblotting in 339 serum samples from patients with PCa and benign prostatic hyperplasia (BPH), and in normal human sera (NHS). The expression of RalA in prostate tumor tissues was evaluated by immunohistochemistry (IHC) in tumor microarrays. The autoantibody level to RalA (median) in NHS was significantly lower than in PCa (0.053 vs 0.138; *P* < 0.001) and BPH (0.053 vs 0.132; *P* < 0.005) groups. The circulating anti-RalA autoantibody could distinguish PCa patients from normal individuals with the area under the receiver operating characteristic (ROC) curve (AUC) performing at 0.861, with sensitivity of 52.9% and specificity of 91.0%. Elevation in serum immunoreactivity was observed in PCa patients after radical prostatectomy. The combined use of both anti-RalA autoantibody and PSA showed a significantly higher discriminatory ability compared with either of those markers alone. RalA protein expression was detected by IHC in 85.3% of tumor tissues from PCa patients, but without significant difference compared to BPH or normal control tissues. Together, our study shows the additional benefits of anti-RalA autoantibody as a potential serological biomarker for PCa, particularly in patients with normal PSA, and further demonstrate the utility of biomarker combinations in the immunodiagnosis of PCa.

## INTRODUCTION

Prostate cancer (PCa) is the most frequently diagnosed cancer (28%) and the second leading cause of male cancer deaths (10%) in the U.S., with an estimated 220,800 cases and 27,540 deaths in 2015 [1]. PCa also disproportionately affects African-American men, who exhibit a significantly higher incidence and mortality from this malignancy than men from other ethnic and racial groups [1]. PCa exhibits a range of clinical behavior, from a slow-growing tumor of little clinical significance to aggressively metastatic and lethal disease [2]. Therefore, the prevention and early detection of PCa, and the identification of patients that are likely to develop aggressive tumors, are issues of major concern in the fight against this malignancy. Previous studies have shown that patients with cancer, including PCa, produce autoantibodies against tumor-associated antigens (TAAs) suggesting that such autoantibodies could have clinical value in early cancer diagnosis and in monitoring cancer prognosis [3–7]. Currently, early detection of PCa using the PSA blood test has increased the proportion of patients with early tumor stage at the time of diagnosis [8]. However, while the sensitivity of the PSA test is exceptional, its specificity, particularly at lower PSA levels, remains controversial [8–11]. Because of these limitations and the heterogeneity of PCa and other diseases of the prostate, there is a critical need for additional, more specific biomarkers that could complement PSA in the early detection and management of PCa.

In recent years, several studies have identified and validated candidate TAAs by profiling the serum autoantibody repertoire from PCa patients. The growing list of TAAs that have been shown to play a role in PCa include prostasomes [4], LEDGF/p75 [12], p53 [13], p90 [14], and 5a-reductase [15]. Our previous findings on anti-TAA autoantibody profiling in cancer have been successfully validated in several independent studies with liver, lung, breast, ovarian, and prostate cancers [16]. It is conceivable that specific autoantibody profiles can be identified with the help for discriminating autoantibody reactivity between cancer patients and control individuals and distinguishing between some types of cancer [17–19].

To increase both the sensitivity and specificity of circulating autoantibodies as biomarkers in PCa patients, we undertook a comprehensive screening of multiple anti-TAA autoantibodies in sera with PCa [20], and then optimized a combination using both anti-RalA autoantibody and PSA in immunodiagnosis of PCa. RAS-like (Ral) proteins, encoded by RalA or RalB, are originally identified on the basis of their sequence similarity to the RAS family of small GTPases [21]. RalA, phosphorylated by Aurora kinase A and other kinases, is a substrate of protein phosphatase 2A (PP2A) Aβ [22]. Evidence indicates that dephosphorylation of RalA is a major mechanism by which PP2A Aβ normally restricts tumor progression, which appears to be a critical step in the Ras-induced transformation and tumorigenesis of human cells [22]. Our recent studies have provided evidence that RalA is overexpressed in hepatocellular carcinoma (HCC), and may have potential as a tumor biomarker in HCC detection [23, 24]. Although the role of RalA in PCa has been noted [25, 26], the immunoreactivity of this protein in PCa patients remains to be investigated. Several human cDNAs encoding candidate TAAs, including RalA, were identified in our previous study with liver cancer sera using the SEREX (serological analysis of recombination cDNA expression libraries) approach [24]. In the present study, we used recombinant RalA as a target autoantigen, and evaluated its immunoreactivity by ELISA and Western blotting in sera from patients with PCa and benign prostatic hyperplasia (BPH), and in normal human sera (NHS). Indirect immunofluorescence (IIF) assay in cell lines and immunohistochemistry (IHC) in tissue microarrays (TMAs) were performed to analyze RalA protein expression in PCa.

## RESULTS

### Prevalence of autoantibody to RalA in PCa, BPH and normal individuals

**Figure 1:**
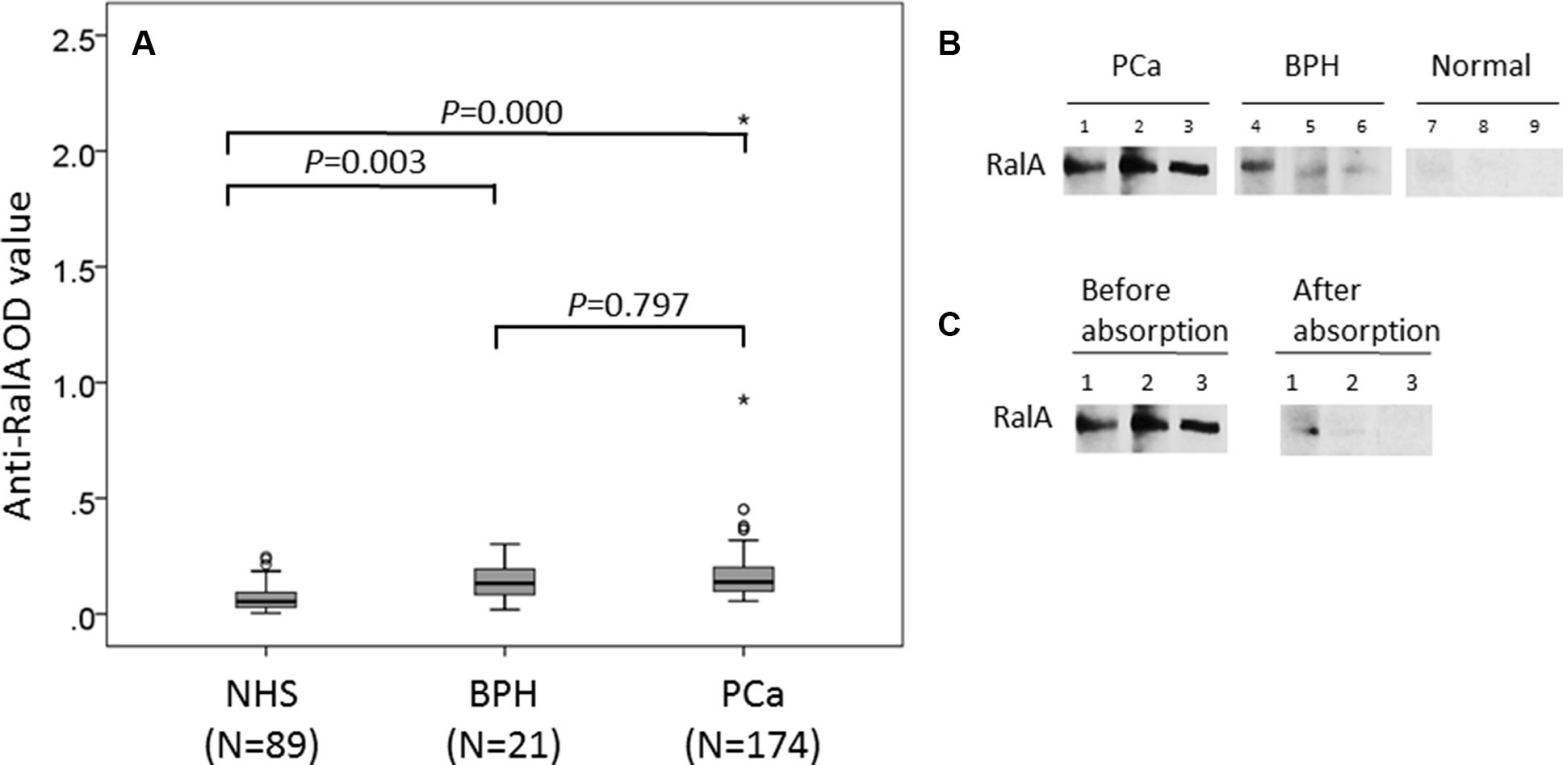
Detection of autoantibodies against RalA in human sera by ELISA and Western blotting analysis (**A**) Autoantibody level to RalA detected by ELISA is expressed as optical density units. (**B**) Western blotting showed the anti-RalA immunoreactivity of representative sera from three patients with PCa (lanes 1–3), three patients with BPH (lanes 4–6), and three normal human subjects (lanes 7–9). The PCa and BPH sera used for Western blotting were from patients that contain antibodies against RalA as detected by ELISA and the ODvalues in ELISA correlated with the intensity of signals in Western blotting. (**C**) The serum anti-RalA immunoreactivity of the PCa patients decreased dramatically after pre-absorption with recombinant RalA protein.

**Figure 2:**
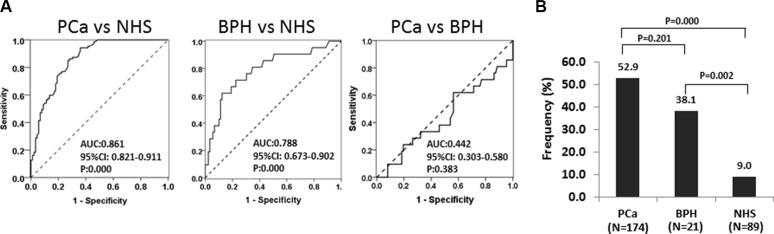
The ROC curves discriminate NHS from PCa and BPH groups of anti-RalA autoantibody (**A**) Receiver operating characteristic (ROC) curve analysis of RalA expression to discriminate the NHS group from the PCa and BPH groups. The area under the ROC curve (AUC) corresponding to the comparisons between pairs of these groups is indicated. (**B**) Frequency of autoantibody responses to RalA in PCa, BPH, and NHS. The frequencies (%) correspond to autoantibody titers exceeding the cut-off value from ELISA. Cut-off value: 0.138 (highest Youden's Index with > 90% specificity).

We first evaluated anti-RalA autoantibody levels in sera from patients with PCa and controls, using the full-length recombinant RalA protein as coating antigen in ELISA. As shown in Figure 1A, among 174 PCa sera, the autoantibody levels to RalA (median) in the NHS were significantly lower than in PCa (0.053 vs 0.138; *P* < 0.001) and BPH (0.053 vs 0.132; *P* < 0.005) groups. Figure 1B showed three representative PCa sera that exhibited positive antibody reaction to RalA in ELISA as well as strong reactivity in Western blotting analysis. Their immunoreactivities were significantly decreased and even disappeared after the pre-absorption with recombinant RalA (Figure 1C). The ROC curves discriminated between PCa and BPH from NHS groups of anti-RalA autoantibody with AUCs of 0.861 (PCa vs NHS) and 0.788 (BPH vs NHS), respectively (Figure 2A). This analysis showed that 52.9% (92/174) of the PCa patients produced autoantibodies to RalA, compared to 38.1% (8/21) of BPH patients and 9.1% (8/89) of normal controls (Figure 2B). In this case, a statistically significant increase in the frequency of anti-RalA autoantibody was observed across these three study groups (*P* for trend < 0.001), although there was no significant difference between PCa and BPH group. The anti-RalA autoantibody as a biomarker for detection of PCa has a sensitivity of 52.9% and specificity of 91.0% relative to normal controls.

### Elevation of anti-RalA autoantibody level in PCa patients after surgical treatment

**Figure 3:**
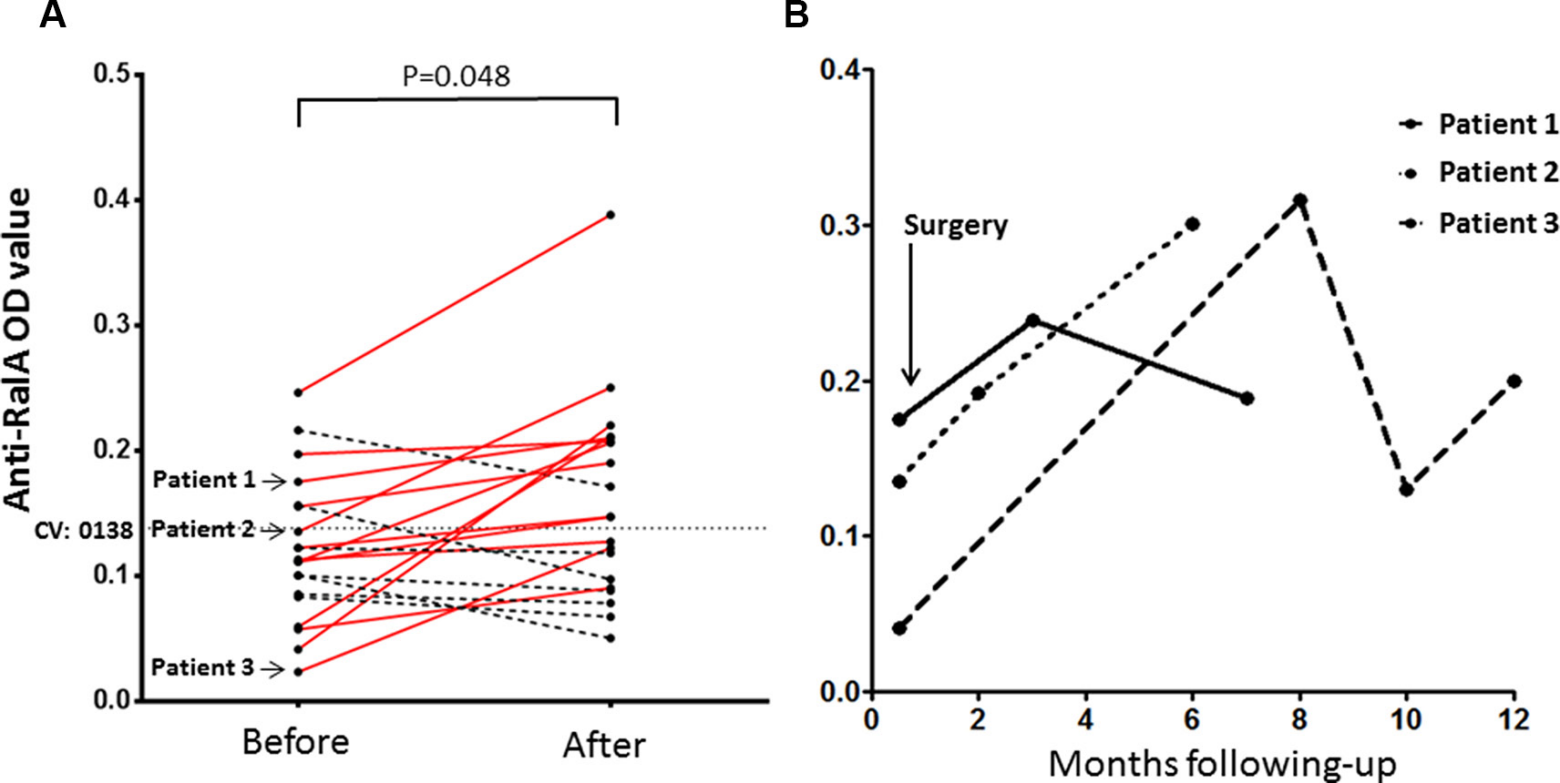
Serial assay of anti-RalA by ELISA in 20 patients with PCa who underwent radical prostatectomy surgery (RP) or transurethral resection of the prostate (TURP) (**A**) The OD values after surgery were the average OD values determined on serum samples that had been collected at a given time point 1–12 months after surgery (mean, 5.5 months). Elevation of immunoreactivity was observed in the most patients (*P* = 0.048, Wilcoxon test). Solid red lines show the elevation of anti-RalA autoantibody and dotted black lines show the decrease of the autoantibody. (**B**) Serial study of anti-RalA autoantibody levels (OD value) during a 1-year period in three representative PCa patients. CV: Cut-off value

Since 55 serial serum samples from 20 PCa patients were obtained at a wide range of time period (ranging 0 to 400 days after surgery), we questioned whether RalA autoantibody levels might change over time after the surgery. As shown in Figure 3, the presence of autoantibodies to RalA was assessed by ELISA over time in the serially collected samples after surgery. The OD value after surgery was the average OD determined on serum that had been collected at a particular time point after surgery during a month period (mean, 5.5 months). Interestingly, elevation of RalA immunoreactivity was observed in most patients (*P* = 0.048, Figure 3A). Serial serum samples were available for 20 patients before and after the surgical resection of prostate tumors, and 11 of these 20 patients were found to have positive results for anti-RalA autoantibody (Figure 3A). We observed 6 patients that showed conversion from negative to positive anti-RalA autoantibody, 4 patients that showed positive titers but displayed increasing immunoreactivities, and 1 patient with decreased titers. Anti-RalA autoantibody titers increased immediately after surgery in three patients (Figure 3B), with patients 1 and 3 showing subsequent autoantibody decreases several months after surgery, which may imply a potential association between changes in anti-RalA autoantibody titers and disease prognosis.

### Immunofluorescence staining of RalA in PCa cells

**Figure 4:**
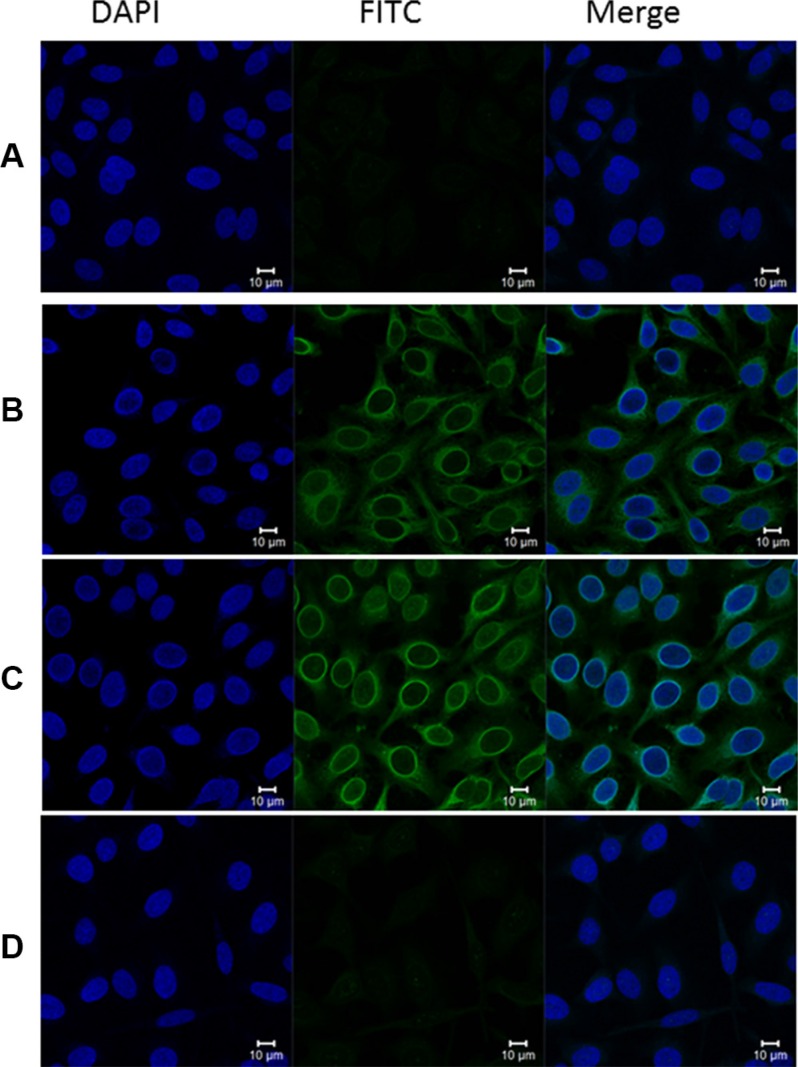
Representative immunofluorescence staining pattern of anti-RalA autoantibody in a positive PCa serum (**A**) Phosphate-buffered saline (PBS) was used as blank control; (**B**) Monoclonal anti-RalA antibody showing a perinuclear IIF staining pattern was used as positive control; (**C**) A representative anti-RalA autoantibody positive PCa serum demonstrated similar perinuclear IIF staining pattern; (**D**) The same PCa serum used in panel C was pre-absorbed with recombinant RalA protein, and subsequently utilized for immunofluorescence assay. The fluorescent signal was abolished by the pre-absorption.

Using IIF microscopy in metastatic LNCaP cells, we screened the anti-RalA positive PCa sera in ELISA to further confirm the specificity of this autoantibody response, and also explore the intracellular location of the RalA protein. Anti-RalA positive PCa sera showed a cytoplasmic staining pattern with more intense staining in the perinuclear regions or partially in intracellular membrane, and this pattern was a similar to that produced by a commercially available monoclonal RalA antibody (Figure 4). Furthermore, the IIF signal was significantly reduced when the sera were pre-absorbed with recombinant RalA. The RalA staining in these cells was consistent with previous studies with other cancer cells [23, 27].

### Expression of RalA in PCa tissues by immunohistochemistry with tissue array

**Figure 5:**
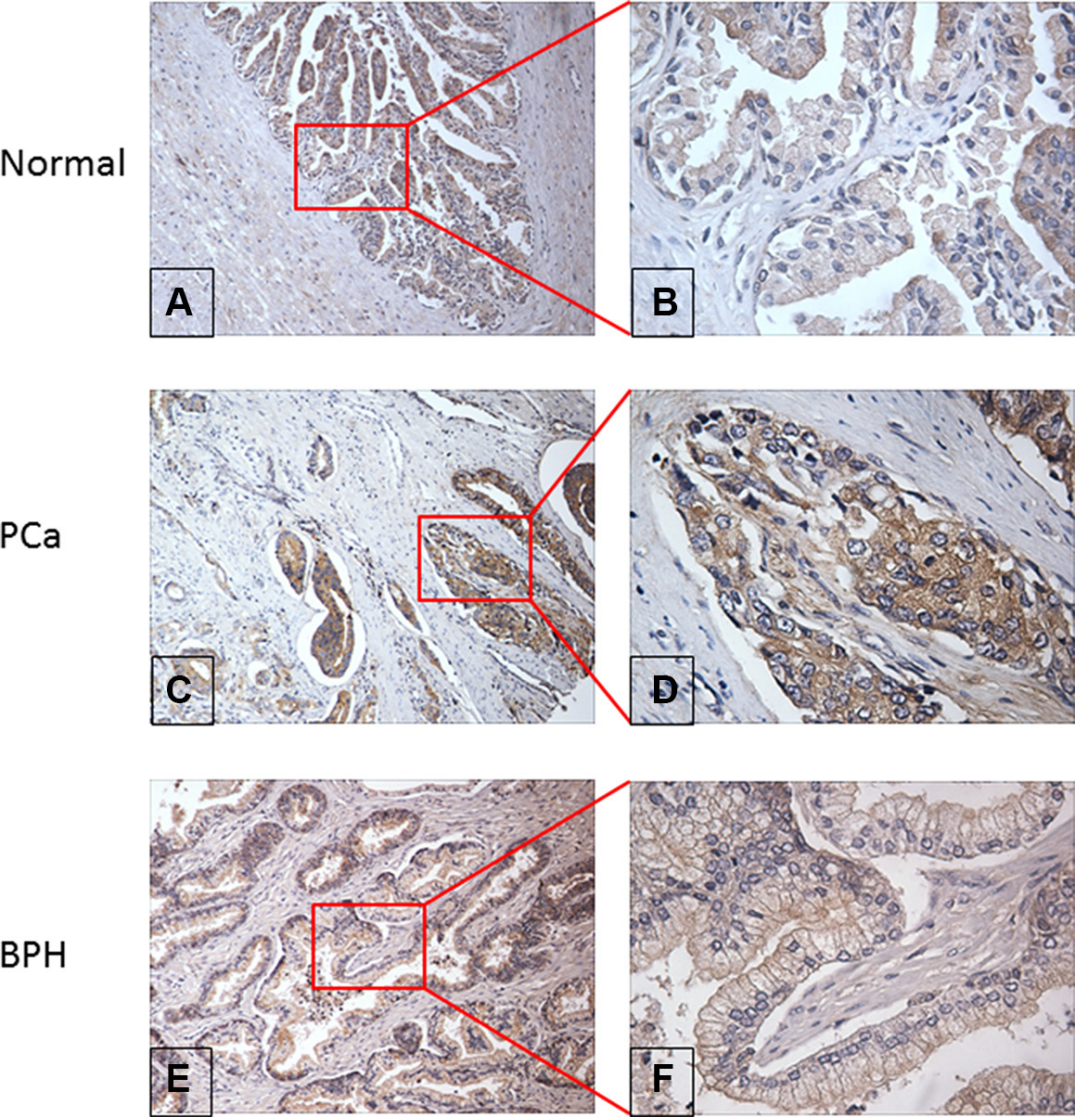
Evaluation of RalA protein expression in normal, PCa and BPH prostate tissue by immunohistochemistry (**A & B**) Moderate positive staining of RalA expression in representative normal prostate tissue at 100× and 400× magnification respectively; (**C & D**) Weak positive staining of RalA expression in PCa tissue at 100× and 400× magnification respectively; (**E & F**) Strong positive staining of RalA expression in BPH tissue at 100× and 400× magnification respectively.

To explore the possibility that RalA is overexpressed in prostate tumors, an expression analysis of this protein in prostate tissue was performed by immunohistochemistry. Tissue microarray slides including 34 PCa tissue specimens, 26 BPH specimens, 6 chronic inflammation (CI) specimens as well as 20 normal prostate tissue specimens, were commercially available for this study. As shown in Table 1, the results indicated that there was no significant difference of the frequency of RalA overexpression in these prostate tissues: PCa (85.3%, 29/34), BPH (61.5%, 16/26), CI (66.7%, 4/6) and normal prostate tissue (78.6%, 11/14). To investigate the possible relationship between tumor stage and RalA expression, we analyzed the clinical characteristics of 34 prostate tumor specimens from the same tissue array that had information on pathology grade, clinical stage, and Gleason scores. Table 1 shows that the frequencies of RalA positive staining in pathology grade 1–2 and 3–4 were 84.2% (16/19) and 85.7% (12/14), clinical stage I–II and III–IV were 92.3% (12/13) and 84.2% (16/19), Gleason scores 2–6 and 7–10 were 83.3% (15/18) and 92.3% (12/13), respectively, without statistically significant difference. Figure 5 shows representative normal, PCa, and BPH tissues with positive immunostaining for RalA.

**Table 1:** Expression profile of RalA in PCa, BPH, CI and normal prostate tissue

Type of tissues	No. tested	Median of IHC score	Frequency of RalA overexpression (%)	*P*
Normal	14	4	11 (78.6%)	
CI	6	5	4 (66.7%)	0.613^a^
BPH	26	4	16 (61.5%)	0.316^a^
PCa	34	5	29 (85.3%)	0.887^b^
Pathology Grade				
1–2	19	4	16 (84.2%)	1.000^a^
3–4	14	5	12 (85.7%)	
Clinical Stage				
I–II	13	5	12 (92.3%)	0.629^a^
III–IV	19	5	16 (84.2%)	
Gleason Score				
2–6	18	4	15 (83.3%)	0.621^a^
7–10	13	5	12 (92.3%)	

Abbreviations: BPH, benign prostatic hyperplasia; CI, chronic inflammation; PCa, prostate cancer. RalA overexpression: IHC score ≥ 4.

a Fisher's exact test.

b Chi-square test.

### Simultaneous use of PSA and anti-RalA autoantibody as markers in PCa detection

Currently, PSA is commonly used as a serum biomarker for blood-based PCa screening. The PSA test has insufficient sensitivity/specificity and often yields false positives leading to unnecessary biopsies, and fails to detect a significant number of PCa cases at the recommended threshold of 4 ng/ml [28–30]. In the present study, 35 PCa patients were available for further PSA analysis. The sensitivity of simultaneous use of PSA and anti-RalA autoantibody as serological biomarkers in PCa detection is shown in Table 2. Eighteen of 35 (51.4%) PCa sera had abnormal serum PSA (≥ 4 ng/ml), which was consistent with a previous report [8]. Interestingly, there were 10 of 17 (58.8%) PCa sera with normal range of PSA levels (< 4 ng/ml) that were positive for anti-RalA autoantibody. When both anti-RalA autoantibody and PSA were simultaneously used as diagnostic markers, 28 of 35 (80.0%) PCa patients could be correctly identified.

**Table 2:** Sensitivity of combined use of both PSA and anti-RalA autoantibody in prostate cancer detection

PSA level (ng/ml)	RalA		Total
	+	–	
≥ 4	9 (A)	9 (B)	18
< 4	10 (C)	7 (D)	17
Total	13	22	35

Sensitivity (%) of PSA = A + B/(A + B + C + D) = 18/35 = 51.4%.

Sensitivity (%) of RalA = (A + C)/(A + B + C + D) = 19/35 = 54.3%.

Sensitivity (%) of combined both PSA and RalA = (A + B + C)/(A + B + C + D) = 28/35 = 80.0%.

RalA positive individuals with PSA < 4 ng/ml = 10/C + D = 10/17 = 58.8%.

## DISCUSSION AND CONCLUSIONS

Compelling evidence has demonstrated that the production of autoantibodies to a unique group of autologous cellular antigens called TAAs constitutes an integral component of the anti-tumor immune response in cancer patients [31–33]. The present study screened 174 PCa sera as well as 55 serial sera from 20 PCa patients, and also tested sera from two control groups, including 21 BPH patients and 89 normal human individuals with no clinical evidence of PCa. The ELISA data indicated that autoantibodies to RalA were present in 52.9% of PCa sera and in 38.1% of BPH sera, which were significantly higher than that in normal individuals (9.0%). The results of IIF microscopy analysis also confirmed the specificity of autoantibody response to RalA in PCa sera.

Our previous studies have shown that once a TAA is identified, the combined use of various immunoassays (e.g. ELISA, IIF and immunoblotting) is required to enhance the detection of specific autoantibodies to that particular TAA in the cancer of interest and determine more accurately the frequency of these autoantibodies in patient sera [12, 18]. Thus, we hypothesized that the autoantibody response to RalA in PCa could be indicative of aberrant expression of this protein in PCa tissues. Due to the difficulty in obtaining PCa tissue specimens from the same patients producing anti-RalA autoantibodies, we had to use a commercial PCa tissue microarray slides to evaluate the expression of RalA. The results indicated that RalA was present in both non-tumor prostate tissues (healthy donors and BPH) and malignant prostate tissues. However, as indicated by the IHC scores, the expression levels of RalA in the PCa specimens appeared to be similar to those in the benign prostate tissues, which implies that RalA may not be associated with prostate tumor progression. This remains to be confirmed in a more comprehensive analysis of RalA expression in normal prostate, prostatitis, BPH and prostate tumors with different stages. Most of the tumors showing higher score of RalA expression were at low grade or stage; however, because the number of tumor specimens provided in the tissue array was relatively small, it was not possible to establish a statistically significant correlation between RalA expression and tumor grade or stage. Our IHC results are also consistent with a report from Smith et al. showing that Ral expression levels in PCa did not correlate with Gleason score [26].

Although the role of RalA in PCa metastasis has been well characterized [34], the immunoreactivity of this protein in PCa patients is poorly defined. Given that the data in the present study showed that the frequency of serum autoantibodies to RalA was significantly higher in PCa (52.9%) sera compared to NHS (9.0%), it could be speculated that RalA function or expression during PCa tumorigenesis stimulates an immune response. These results suggest that RalA could be used as a potential tumor marker in PCa detection or as a candidate antigen for the development of PCa immunotherapies. Whether higher circulating anti-RalA autoantibody is an accelerator or attenuator of PCa, or merely an indicator of the presence of a prostate tumor (in the case of PCa) or prostate inflammation (in the case of BPH), remains uncertain. The statistically increased expression of anti-RalA autoantibodies in PCa and BPH relative to NHS, although there was no significant difference between the PCa and BPH groups, suggest that these autoantibodies may arise as the result of RalA upregulation in the context of chronic inflammation of the prostate, which is common to both PCa and BPH [35, 36]. It would be of interest to determine if these autoantibodies are also elevated in patients with prostatitis, a condition that is considered as a frequent precursor to PCa given its role in promoting an inflammatory prostate microenvironment during early prostate carcinogenesis leading to proliferative but atrophic epithelial cells with notable inflammatory infiltrates [37, 38].

Radical prostatectomy is one of the most common treatments for PCa and generally provides excellent cancer control [39]. However, approximately 35% of patients undergoing this procedure will develop biochemical recurrence within 10 years after surgery, which often gives rise to more aggressive, metastatic tumors [40]. Given that autoantibodies in PCa patients may be sensors or sentinels of events associated with prostate tumorigenesis [18], it would be tempting to speculate that increases in anti-RalA autoantibody levels after radical prostatectomy may reflect changes in the expression or activity of this protein in the patients. Our cohort study with serial serum samples indicated that there was significant increase of anti-RalA autoantibody level in several PCa patients after surgical treatment. This could be possibly explained by tumor-induced immunosuppression prior to surgery, which involves reduced immune functions in tumor-bearing individuals as a fundamental mechanism allowing tumors to escape immune destruction [41]. Consequently, it is likely that radical prostatectomy reverses such suppression by reducing the levels of immunosuppressive factors, thereby allowing the immune response to recover in the absence of inhibitory cytokines [42]. If this is the case, then immunosuppression may recur as metastatic lesions grow and inhibitory cytokine levels increase, becoming more severe as metastatic tumor burden increases [42]. This scenario could provide another possible understanding of why anti-RalA autoantibody titers increased dramatically immediately after radical prostatectomy in some patients, followed by reduced anti-RalA autoantibody level several months later. However, whether the changes of anti-RalA autoantibody titers have an association with early-onset metastatic disease following primary tumor resection is unclear given the retrospective nature of our study. Additionally, comprehensive studies on the biology of RalA in prostate cancer progression and metastatic castration-resistant PCa are guaranteed to better understand the significance of this autoantibody.

Serum prostate specific antigen (PSA) is the most commonly used PCa biomarker in clinical practice. However, its low specificity (50%) may produce a high number of false positives [43]. There is evidence demonstrating that cancer autoantibody profiling using a panel or mini-array of TAAs can significantly improve distinguishing PCa-associated autoantibodies from autoantibodies triggered by other conditions [44–47]. Our pioneering studies with TAA mini-arrays indicated that an optimal combination of multiple TAAs yields increased sensitivity and specificity for immunoserological diagnosis of PCa [17, 19]. In the current study, a novel assay platform of anti-RalA autoantibody plus PSA was established to improve the sensitivity of PCa detection to 80.0%, which indicates that anti-RalA autoantibody might be an independent marker but also a supplementary serological marker that could enhance the detection of biochemical recurrence in PCa [48]. Although the possible mechanisms underlying the production of anti-RalA autoantibodies remains poorly known, it is likely that they are related to dysregulated function of this protein or alteration of its molecular structure or location in the context of a pro-inflammatory microenvironment [49]. Thus, further studies are clearly needed to verify the association between autoantibodies to RalA and individuals at high-risk of developing prostate cancer or disease recurrence after treatment by cross-validating in larger and broader patient cohorts. Moreover, efforts should be aimed at optimizing both the sensitivity and specificity of serum autoantibodies as PCa markers by expanding TAA arrays to include antigens that might be more uniquely associated with PCa.

In summary, to the best of our knowledge, this is the first study to report that certain patients with PCa produce autoantibodies to the RAS-like protein RalA and that this protein is notably highly expressed in prostate tissues. Furthermore, anti-RalA autoantibody combined with PSA signatures did detect additional patients than either alone, suggesting that this combination may aid early detection of prostate cancer. Moreover, anti-RalA autoantibody level was potentially associated with disease prognosis in this subset of patients, implying that this marker may be a further tool not only for diagnosing PCa but also for disease prognosis. Taken together, our results suggested that the autoantibody to RalA might be considered as a potential serological biomarker for PCa.

## MATERIALS AND METHODS

### Serum samples

Sera were obtained with informed consent from randomly selected patients with PCa (*n* = 174), at Loma Linda University Medical Center. In addition, the samples drawn several days before and after surgical operation of 35 patients with early stage PCa (I/II stage) were obtained from the serum bank in the Autoimmune Disease Center at The Scripps Research Institute (La Jolla, CA). And 20 of them have serial serum samples collected at least two to four samples obtained at different time points. The PSA levels in the sera from 35 patients were available. Among them, 51.4% (18/35) sera had abnormal PSA level (≥ 4 ng/ml), whereas 17 (48.6%) had normal level (< 4 ng/ml). Since this study was originally designed to evaluate the serum autoantibody repertoire in PCa, two control groups were also selected for the study. The first group consisted of sera drawn from 21 BPH patients in Loma Linda University Medical Center and Faculty Medical Offices. The second group consisted of NHS (*n* = 89) obtained from the serum bank by the Autoimmune Disease Center at the Scripps Research Institute, La Jolla, which were collected from adults during annual health examinations in people with no clinical evidence of PCa. All samples were collected, processed and stored in a similar fashion. This study was approved by the Institutional Human Subject Review Boards of the University of Texas at El Paso and collaborating institutions.

### Cell lines

LNCaP prostate cancer cells (brain metastasis) were purchased from the American Type Culture Collection (Manassas, VA). Cells were maintained in a humidified atmosphere with 5% CO_2_ at 37°C using the supplier's prescribed RPMI 1640 (Invitrogen) medium supplemented with 10% FBS and penicillin/streptomycin.

### Preparation of recombinant RalA

Recombinant RalA was prepared and used previously [23]. Briefly, a pET-28a plasmid with N-terminal 6x histidine and T7 epitope tags, encoding full-length cDNA encoding RalA amplified by PCR from a human EST (expressed sequence tag) clone (#BM560822), was transformed into *E. coli* BL21 (DE3) cells and the IPTG-expressed recombinant RalA protein was purified using nickel column chromatography. The purity and integrity of recombinant RalA were further analyzed by electrophoresis on SDS-PAGE, and the protein was identified by Western blotting by anti-histidine antibody and commercial monoclonal anti-RalA antibody.

### ELISA

ELISA procedures were performed essentially as indicated in previous studies [7, 16, 17, 23]. Briefly, purified recombinant RalA protein was diluted in phosphate-buffered saline (PBS) to a final concentration of 0.5 μg/ml and coated onto a 96 well microtiter plate (Dynatech Laboratories, Alexandria, VA). Human sera diluted at 1:200 were incubated in the antigen-coated wells. Horseradish peroxidase (HRP)-conjugated goat anti-human IgG (Caltag, Burlingame, CA) and the substrate (1 mg/ml 2,2-azino-bis[3-ethylbenzthiazoline-6-sulfonic acid] with 0.005% hydrogen peroxide in citrate buffer, pH 4.6) were used as detecting reagents. The optical density (OD) was measured at 405 nm using an automated plate reader (SpectraMax 190; Molecular Devices, Sunnyvale, CA). All serum samples were assayed in duplicate and all positive sera were further confirmed by Western blotting.

### Indirect immunofluorescence (IIF) microscopy

LNCaP cells grown on coverslips were washed with PBS, fixed for 5 min at −20°C in 100% methanol, permeabilized for 3 min at −20°C in 100% acetone, and stained with a highly specific human anti-RalA serum (1:80 dilution) which showed positive reactivity in ELISA. Monoclonal anti-RalA antibody at 1:2,000 dilution was used as a positive control. NHS and PBS were used as negative and blank controls, respectively. After incubation of primary antibodies for one hour in a humidity chamber, followed by washes with PBS, the FITC-conjugated secondary goat-anti-human IgG (Caltag Laboratories, San Francisco, CA) was applied at 1:200 dilution. The slides were counterstained with DAPI (4′,6-Diamidino-2-phenylindole dihydrochloride) and examined in a confocal laser-scanning microscope (LSMS PASCAL, Carl Zeiss GmbH, Jena, Germany). All images were acquired under identical conditions. Ambiguous results were considered negative.

### Serum absorption for immunological tests

Sera with the same dilutions used in Western blotting or IIF microscopy were pre-absorbed with recombinant RalA protein at a concentration of 0.03 ug/ml by overnight incubation at 4°C, followed by brief centrifugation and analysis of the supernatant by Western blotting and IIF microscopy.

### Immunohistochemistry (IHC)

Immunohistochemical study was performed using a commercially available prostate disease spectrum tissue microarray (TMA) (catalog no.: PR8011, US Biomax, Inc.) consisting of 34 PCa tissues, 26 BPH, 6 chronic inflammation, and 14 normal tissues. The tissues were deparaffinized, endogenous peroxide was blocked in 3% hydrogen peroxide in methanol, and microwave antigen retrieval was done using a citrate-based antigen retrieval solution (BioGenex, San Ramon, CA). Blocking was done using 1.5% normal horse serum and TMA slides were incubated with monoclonal anti-RalA antibody at concentration (1:2,000 dilution). Biotinylated secondary antibody, ABC (Avidin: Biotinylated enzyme Complex), and DAB (3,3′-diaminobenzidine) substrate were used as detecting reagents (Vector Laboratories, Burlingame, CA). The tissues were counterstained with hematoxylin, fixed by Scott's solution and dehydrolyzed with different concentration of ethanol and Citrisolvent. Finally, the slides were mounted with permount mounting medium and observed under brightfield microscopy (Leica DM1000, Germany). Briefly, five representative 400X magnification fields for each patient were randomly selected for histology evaluation. Positive rate and staining intensity were used to describe the expression based on the number and staining intensity of positively-stained cells in the tissue samples. The sum of positive rate core and staining intensity score was used to estimate the antigen expression in each sample, in which the final score, < 4 was defined as low/negative expression, whereas ≥ 4 was defined as high expression [50].

### Statistical analysis

Data regarding the different immunoreactivity of the sera were summarized by median and compared using the non-parametric Kruskal-Wallis tests, and pair-wire compare between every two groups was further analyzed by Wilcoxon test with Bonferroni adjustment. Chi-square test or Fisher's exact test was used to compare the difference of autoantibody frequency between groups. The sensitivity and specificity was evaluated using the receiver operating characteristic (ROC) curve analysis, leading to estimates of the area under the curve (AUC), with 95% confidence intervals. The optimal cut-off value (CV) for the optical density (OD) of an ELISA was determined by ROC analysis of the maximum Youden index when the specificity was > 90.0% [51]. Statistical analysis was carried out in SPSS software, version 19.0. Differences were considered statistically significant by significant level 0.05.
